# Book Notices

**Published:** 1866-04

**Authors:** 


### EDITORIAL.

### BOOK NOTICES.

*The Ophthalmoscope as a Means of Investigating the Diseases of the (Brain) Nervous System.*

In the last volume of the "Royal London Ophthalmic Hospital Reports," (1865) is the first part of a most interesting paper, by Dr. J. H. Jackson, Assistant Physician to the National Hospital for Epilepsy and Paralysis, and to the London Hospital.

The paper is worthy the most careful attention of every physician. The author modestly disclaims all pretensions to skill or experience as an oculist, and yet has evidently made himself acquainted in no imperfect manner with the practical use of the ophthalmoscope, in aiding him as a physician to study the diseases of the nervous system. There is certainly reason why a physician, especially interested in these diseases, should be a good ophthalmoscopist; for with the ophthalmoscope we are able to look upon the end of the optic nerve, upon and through the retina, and watch the abnormal changes as they take place in these structures, so near the brain and so intimately connected with it in their vascular system, in fact the only nervous tissues, spread out so wonderfully to our view. We can give but a few passages from this interesting paper:

"I wish to repeat what I said in a former communication—that I write as a physician, and not as an ophthalmologist. I hope this will be remembered if I am thought to treat amaurosis too diffusely in its general bearings as a symptom, or less precisely in its special one as an eye-disease. To expect from a physician a wide and advanced knowledge of a department of medicine now so special and so rapidly advancing as ophthalmology, would be, to expect what is impossible. My object in writing this article is, simply that I may contribute from my own field of clinical work a few observations on a class of symptoms, the meaning of which it is most desirable to make out for the sake of advancing our knowledge of diseases of the nervous system."

"What we generally see with the ophthalmoscope in amaurosis *in cases of disease of the nervous system*, for instance, in amaurosis complicating epileptiform convulsions, hemiplegia, etc., is a white condition of the optic discs—sometimes called the optic papilla or end of the optic nerve—white atrophy. But there are at least two kinds of white atrophy: (1st) One in which the nerve gradually whitens; and (2d) another in which it becomes white after certain acute changes. The first of these might be called nerve atrophy, and the second cerebral atrophy, but I will speak of them as No. 1 and No. 2 respectively. In No. 1 the disc is more or less white, round, and well margined, and the central vessels do not always seem to be diminished in size. In No. 2 the edge of the disc is not well defined. It sometimes gradually merges into the fundus, and is sometimes ragged. The vessels are generally diminished in size, and there is a scattering of pigment in the neighborhood of the disc."

"Another reason why the physiology of amaurosis is still obscure, is that in acute cases of cerebral diseases, in which we have the patient under our observation from first to last, the amaurosis is often overlooked. This no doubt will seem a strange statement, but it is quite true, and it is easily accounted for. I am convinced that in all cerebral cases we should accept our patients' statements as to their power of sight with great caution, or rather we should interpret them cautiously, since it is the intra-ocular changes that we run most risk of overlooking. For not unfrequently in the early stage of cerebral amaurosis, whilst there are to be seen most marked changes in and around the optic disc, with for instance considerable effusion of blood, the patient will complain of but slight dimness, and will read small type. I had under my observation a few months ago a patient who used to read the newspaper every day, and who read small print easily in my presence, and yet had the ophthalmoscopic appearances of the acute stage of No. 2 well marked, the effusion of blood around the discs being considerable. This patient subsequently became almost quite blind, his sight eventually failing rather suddenly. He died, and I found at the *post-mortem* examination a tumor at the base of the brain."

"In cases like those just mentioned, the sight sometimes fails suddenly, the ophthalmoscopic appearances remaining nearly the same, *i. e.*, so far as I have been able to estimate them. Now it is most important to keep this in mind, or we might otherwise get a wrong date for the appearance of amaurosis as a symptom. It is well to observe, also, that with the above mentioned ophthalmoscopic signs of fixed damage to the eye, the sight fails occasionally. The patient makes some such remark as this, "I come over blind now and then." It is difficult to account for such variations, but the difficulty is no greater here than in many other organic diseases of the nervous system, which so frequently show an element of periodicity more or less rough in their symptoms. Thus neuralgic pain from fixed organic disease, say of the Casserian ganglion, comes on in paroxysms. To magnify the fact of periodicity in this kind of amaurosis, by saying that periodicity is an element in all diseases of the nervous system, does not, of course, make the cause of the phenomena more evident, but it has the advantage of enlarging our field for studying an important law. At one time I thought these variations of importance as regards the question of reflex amaurosis. I supposed they indicated that the "contraction of the vessels" was intermittent. The most marked case of intermittent vision I have seen was in a patient who had incomplete amaurosis, with a disc of the first kind. In the acute stage of No. 2, it seems to be more often due to mechanical causes, as stooping, than to quasi-physiological ones, like contraction or relaxation of the vessels.

"To be quite certain, we ought in every case of disease of the nervous system, when our patient complains of but the slightest defect of sight, to examine the eyes with the ophthalmoscope, and even when the effect is said to be but an occasional failure, we ought not to omit to do this, as such failures do not always indicate loss of power of accommodation merely, nor what I have spoken of as Epilepsy of the Retina.

"In the out-patient's room at the Hospital for Epilepsy and Paralysis, and at the London Hospital, there is a gas-lamp for these examinations. (This lamp serves also for the laryngoscope.) When everything is thus ready, it takes very little time to make an ophthalmoscopic examination. It is quite a different thing when one has to make an examination at the home of one of our hospital patients, especially when he is very ill, awkwardly placed in bed in a confined room, and where the only light to be had is, that of a candle, held by the patient's pillow.

"I have known a physician to be in doubt whether a child was blind or not. It was certainly difficult to tell by ordinary tests, as the child was lying in bed, was paralyzed, and was very fretful. Had the ophthalmoscope been used, there need have been no doubt as to whether there were intra-ocular changes or not. I am convinced that patients frequently die blind without any knowledge of it on the part of their friends. Indeed, the record of an acute case of cerebral disease must be considered to be imperfect, if the eyes have not been examined with the ophthalmoscope."

"*It is, I submit, imperative in all cases of severe cerebral disease, at all events in cases of an acute kind, to examine the eyes with the ophthalmoscope, whether the patient complains of defect of sight or not.* I do not say that it is important as a guide to treatment, but it is absolutely necessary if cases are to be used as materials for scientific purposes. In short, in many cases we are bound to record, not only what the patient tells us, but what we see with the ophthalmoscope."

"Moreover, the above note shows that, early in a case, there may be no marked ophthalmoscopic signs to give enough definiteness to "dimness of sight" attending severe headache, to enable us to recognise in it a special symptom of cerebral disease. Now I by no means wish to imply that "dimness of sight, with a headache is a serious thing," but that it is always imperative to view this series of symptoms most carefully, and especially if the pain be severe, or unusual, and, above all, if attended by urgent, purposeless vomiting. Without coming to any positive conclusion, either to alarm our patients or to fetter our own therapeutical proceedings, we certainly ought to keep our minds open to the fact that such symptoms do sometimes herald severe and often fatal cerebral disease.

"Severe headaches are, I think, too frequently, especially if the patient be subject to them, put down to gastric or abdominal disease. Now I think it is at least, just as likely that severe pain in the head is due to disease within the head, as that pain in any other part is due to disease of that part. And although the instances may be rare, I repeat that we do know that 'bilious' headaches sometimes herald severe brain disease. Certainly, pain in an unusual position, as at the vertex or in the temples, should receive most careful attention and wide consideration."

Thirteen cases of nervous diseases, principally cerebral, in which there were amaurotic symptoms, are related, as illustrating the importance of the subject. We look with interest for the remainder of Dr. Jackson's paper.

---

*Intramural Interments in Populous Cities, and their Influence upon Health and Epidemics.* By JOHN H. RAUCH, M. D., Chicago. 1866. Pp. 68.

This interesting pamphlet appears at a time which insures its favorable reception by a community already awakened to a sense of the necessity of hygienic reform. It presents a concise historical retrospect of the customs pertaining to sepulture, from the earliest antiquity to the present epoch. This is followed by a scientific exposition of the influences prejudicial to health which originate in the putrefaction of animal matter, and the essay is concluded by an application of the doctrines thus deduced to the existing state of facts in Chicago.

Turning from these pages to the chronicles of the ancient world, we are tempted for the moment to believe that the boasted civilization of the nineteenth century after Christ, is below the mark of the nineteenth century before Christ. One cannot but dwell with pleasure upon the age of Roman greatness, when the seven-hilled city was drained by a system of sewers which, though commenced at a period so distant that the date of their construction is lost among the myths of antiquity, were built in a manner so perfect, that even to our own time hardly a single stone has fallen from its place: magnificent conduits, actually navigated by the inspectors; thoroughly cleansed by the unfailing torrents which poured from the distant Appenines through many an aqueduct, the work of wealthy noblemen by whom the public works of the city were undertaken without expense to their fellow-citizens.

But it is to an age still more remote than the Roman that we must look for the most perfect system of sanitary government that the world has ever known. The pages of Herodotus describe an ancient civilization on the banks of the Nile, which will never cease to excite the wonder of mankind. The Egyptian race seem to have passed through none of the progressive stages which have marked the growth of other nations, but they appear upon the stage of the world with a complete system of religion, of laws and of customs which no succeeding generation could improve. The story of those ancient dynasties and the pomp and splendor of the Egyptian hierarchy, are familiar as the annals of our own people, but the care with which the hygienic interests of the inhabitants were guarded may not be so generally known. Many of the requirements of the Mosaic law were merely intended as sanitary precautions, borrowed from the customs of the people by whom the Israelites had been held in bondage. Circumcision, abstinence from pork, the laws pertaining to the purification of the sexes, were all enforced by the Egyptian priesthood long before the days of Abraham. So also with respect to the burial of the dead: it was not sufficient that the remains of human beings should be embalmed and conveyed to the catacombs, but no animal was allowed to remain above ground after life became extinct. The bodies of cattle were interred until the flesh was consumed, and then the bones were removed with sacred rites to certain appointed receptacles, remote from the habitation of men. Dead cats, dogs and birds were all embalmed, and their mummies were consigned to the catacombs. No country was ever in a condition more cleanly and salubrious than was Egypt, before the invasion of Grecian licence and corruption. Nor were these sanitary measures difficult to sustain. The cunning priests invested all their requirements with the sanctity of religion; consequently, when the embalming of a cat or the sepulture of an owl was considered a rite most essential to procure the favor of the Deity, it was easy to enforce a law which might otherwise have been most obnoxious. As the age of superstition, however, passed away, and as men discovered that these rites possessed no real religious merit, they gradually fell into disuse; a whole nation was no longer to be seen, for three days of each month, taking clysters, emetics and purgatives; the dead bodies of the lower animals were slipped into the sacred Nile, without fear of mortally offending the divinity of the stream; and the Egyptians gradually lapsed into that state of filth and squalor which characterizes the majority of mankind.

Delivered from the fetters of superstition and despotism, the world has retrograded in respect of all sanitary observances. Nothing less than the most powerful coercion is sufficient to enforce the habit of cleanliness, and since experience has shown that the ordinary forms of government are insufficient to protect a civic population against itself, and it is no longer possible by investing the subject with the sanctity of religion, to appeal to the strongest motives of the human soul, but one resource remains. The care of the public health must be confided to a commission endowed with despotic powers, and independent of all political control. The politicians and the free people have failed to secure the best interests of the world; the era of priestly dominion seems to be no longer possible; the whole matter must be confided to the physicians, else every city will become what New York has been and what Chicago is fast approaching.

We cannot too cordially commend the essay of Dr. Rauch to the general public as well as to the medical profession. His remarks upon cholera are well timed and eminently practical. If the pestilence should invade our country, we trust that his admirable idea of quarantine hospitals on the lines of railway outside of the town will be acted upon by the authorities who have the matter in charge.

---

**A liberal price will be paid for the following Nos. of the Chicago Medical Journal: Nos. 1 and 4, of Vol. 8, whole series. Address Editors Journal.**

### CODEIA.

We do not presume in these few lines to afford the readers of the Journal any new ideas regarding Codeia. Its peculiar effects, as shown by experiments, are sufficiently well described by several authors. We feel certain, however, that many physicians, from disuse of the remedy in consequence of its high price, have neglected to prescribe it in cases where it may be used with great advantage. And it is here our object simply to urge the trial of Codeia as an anodyne, where it is known by experience that other forms of opium produce very unpleasant effects. We have recently observed the most happy influence of the remedy upon four patients, who often require anodynes for different painful affections, and who always suffered extremely from the secondary effects of morphine and opium however given.

The dose of Codeia may vary, according to circumstances, from gr. $\frac{1}{3}$ to gr. j. It is worthy of notice that the system becomes sooner accustomed to large doses of this anodyne than to corresponding portions of morphine.

---

The contribution on the use of Tannin in pruritus vulvæ, page 560 of the last volume of the Journal, should have been accredited to Dr. G. F. Keiper, of Quincy, Indiana. In each of the last two lines of the page, the quantity should be ℥j, instead of ʒj.

---

**Dr. H. M. LYMAN has removed his Office to 169 Dearborn Street, opposite the Post Office.**

---

WANTED TO PURCHASE, **by a Physician, a small stock of drugs, with an established trade, in a flourishing town on a railroad, in Illinois. Those wishing to sell can address Dr. Z. P. H., care Dr. R. M. Lackey, 169 South Dearborn Street, Chicago.**

---

**A Physician recently mustered out of the U. S. service, desires an eligible location in Illinois. Physicians desiring to sell out, please address, stating price, etc., Dr. Rob't Miller, care of Dr. R. M. Lackey, 169 South Dearborn Street, Chicago.**

